# Conceptualization From the Sensors to Suicide-Related Outcomes: Scoping Review Based on Layered Hierarchical Sensemaking Framework

**DOI:** 10.2196/98616

**Published:** 2026-08-13

**Authors:** Sohee Kim, Jinyeong Kim, Wai Tong Chien, Tzu Tsun Luk, Yu Zhang, Zhen Yang Abel Tan, Eunju Park, Heejung Kim

**Affiliations:** 1 Department of Nursing, College of Nursing, Yonsei University, 50-1 Yonsei-ro, Seoul, 03722, Republic of Korea, 82 2-2228-3273; 2 Brain Korea 21 FOUR Project, College of Nursing, Yonsei University, Seoul, Republic of Korea; 3 The Nethersole School of Nursing, Faculty of Medicine, The Chinese University of Hong Kong, Hong Kong, China (Hong Kong); 4 Alice Lee Centre for Nursing Studies, Yong Loo Lin School of Medicine, National University of Singapore, Singapore, Singapore; 5 School of Nursing, Peking University, Beijing, China; 6 Division of Sleep Medicine, Peking University People's Hospital, Beijing, China; 7 Yonsei Evidence Based Nursing Centre of Korea: A JBI Affiliated Group, Yonsei University, Seoul, Republic of Korea; 8 Mo-Im Kim Nursing Research Institute, Yonsei University, Seoul, Republic of Korea; 9 Institute for Innovation in Digital Healthcare, Yonsei University, Seoul, Republic of Korea

**Keywords:** suicide, passive sensing, digital phenotyping, smartphones, wearable devices, scoping review, suicide-related outcomes

## Abstract

**Background:**

Suicide is a leading cause of preventable mortality worldwide, with more than 700,000 deaths annually. Although suicidal ideation fluctuates rapidly, conventional risk assessments rely on retrospective self-report collected infrequently, and the detection of short-term suicide risk remains limited. Passive digital sensing using smartphones and wearable devices enables continuous monitoring of behavioral and physiological signals associated with suicide-related outcomes. However, current evidence remains fragmented, without a clear framework for translation into clinically interpretable risk indicators.

**Objective:**

This scoping review synthesized and mapped passive digital markers associated with suicide-related outcomes via the layered hierarchical sensemaking framework (LHSF), which structures information from raw sensor data to high-level behavioral markers. We aimed to illustrate a clinically interpretable mapping of digital markers for suicide-specific digital phenotyping.

**Methods:**

Following Arksey and O’Malley and the PRISMA-ScR (Preferred Reporting Items for Systematic Reviews and Meta-Analyses extension for Scoping Reviews) guidelines, this scoping review was conducted using the population-concept-context framework (population: not restricted; concept: passively collected digital data from smartphones or wearable devices; and context: suicide-related outcomes). PubMed, CINAHL, PsycINFO, and IEEE Xplore were searched for studies published between 2015 and 2025. Studies were included if they (1) collected passive digital data from smartphones or wearable devices, and (2) measured suicide-related outcomes. Narrative mapping was conducted using LHSF to distinguish between low-level features (ie, measurable properties extracted from sensors) and high-level behavioral markers (ie, clinically meaningful constructs interpreted from low-level features).

**Results:**

Of 626 studies identified, 14 (2.2%) met inclusion criteria. Six (42.9%) used predictive modeling, and 8 (57.1%) conducted correlational analyses. Among predictive studies (area under the curve [AUC]=0.56-0.89), a lower heart rate variability predicted an elevated suicide risk in 1 study (AUC=0.89). Of correlational studies, 7 (87.5%) of 8 reported at least one significant association between passive sensor data and suicide-related outcomes. Mapped to the LHSF, low-level features spanned 7 domains, linked to high-level markers, such as autonomic dysregulation, sleep disturbance, social withdrawal, smartphone use patterns, and suicide-related expression. Physiological indicators of autonomic regulation were associated with suicide-related outcomes in all 4 studies examining them and achieved the highest predictive performance (AUC=0.89). Smartphone use metrics were significantly associated in both studies, whereas linguistic (2/3 studies, 66.7%) and location-based features (2/2 studies, 100%) were associated with at least one outcome, with nonsignificant findings for some indicators or studies. Sleep parameters and movement intensity showed few significant associations.

**Conclusions:**

Physiological indicators were associated with suicide-related outcomes across all relevant studies and showed the highest predictive performance (AUC=0.89), followed by smartphone-derived behavioral features. Linguistic and location-based features showed mixed associations, whereas sleep- and activity-related indicators showed few significant associations. Future research should prioritize multimodal data integration, algorithmic refinement, and external validation to strengthen clinical utility in digital suicide phenotyping based on the LHSF.

## Introduction

Suicide is a leading cause of preventable mortality worldwide, with more than 700,000 individuals dying by suicide annually, accounting for approximately 1% of all deaths globally [1,2]. Recognizing its substantial public health burden, the United Nations has included a Sustainable Development Goals (SDG)–specific target (SDG 3.4.2) to reduce the national suicide mortality rate by one-third by 2030. Interviews and self-reported questionnaires are frequently used to evaluate suicide risk, such as suicidal ideation, plans, and past behaviors via standardized instruments [3,4]. However, these measures are typically administered at a single clinical encounter or at intervals and rely on individuals’ recall of experiences over extended periods (eg, the past week or month) [5]. In practice, suicidal thoughts and related risk processes are highly dynamic, with meaningful fluctuations occurring over short timescales. Coppersmith et al [6] found that episodes of elevated suicidal ideation often lasted only 1 to 3 hours on average. An ecological momentary assessment (EMA) study in psychiatric inpatients with depression reported that momentary hopelessness and perceived burdensomeness significantly predicted suicidal ideation at the next assessment [7]. EMA studies indicate that only high-frequency, in situ assessments can capture short-term risk processes [8,9]. Consequently, conventional suicide risk assessments based on retrospective recall and infrequent administration often fail to detect brief but clinically critical risk elevations [8,9].

Researchers have increasingly used digital data as a complementary source of behavioral and psychological information to advance early identification and intervention. Recent advances in information and communication technologies have enabled passive digital sensing via smartphones and wearable devices, which has become a promising tool for continuous monitoring of mental health–related physiological and behavioral changes [10]. These devices capture real-world data on physiological reactions, physical functions, and daily activities, and enable assessments beyond clinical settings with ecological validity. This has fueled digital phenotyping research aiming to infer mental health states from patterns in digital traces [11]. Despite increasing digital sensing research on suicide risk, studies report heterogeneous digital indicators, including physiological (eg, heart rate variability [HRV]), behavioral (eg, physical movement), and smartphone-derived contextual features (eg, text language use).

However, this recent evidence remains inconsistent, and the sensors or digital features most robustly associated with suicidal thoughts and behaviors remain unclear for several reasons. First, the robustness of sensor signals is often insufficient; many passive sensing features, including physiological measures, lack stability across devices or populations and provide limited incremental value beyond traditional self-report measures [12]. Second, individual heterogeneity makes universal indicators difficult to define, as suicide risk is an idiosyncratic process rather than a monolithic state [13]. Although specific markers, such as active midnight smartphone use (11 PM to 1 AM), may predict next-day ideation, other indicators, such as decreased social frequency, remain ambiguous. These indicators may signal crisis for some but represent benign routine for others [14]. Such variability, often referred to as phenotypic equifinality, indicates that similar digital behaviors reflect different risk levels based on an individual’s baseline, complicating standardized interpretation. Third, traditional gold-standard interviews often miss the proximal, transient triggers (eg, acute midnight loneliness or sudden sleep deprivation) that are visible only through “screenomics” data [15], creating a disconnect between real-time digital markers and traditional interviews. Consequently, clear guidance on the sensors or digital features that should be prioritized for reliable monitoring is lacking, limiting the translational potential of digital sensing in suicide or other clinical risk assessment.

Addressing these limitations requires a structured framework that can systematically translate passive sensor data into clinically meaningful suicide-related states. Mohr et al [16] proposed the layered hierarchical sensemaking framework (LHSF), which conceptualizes the transformation from low-level sensor data to high-level behavioral markers. This scoping review aimed to apply the LHSF to (1) systematically map how digital sensor data were transformed into low-level features, high-level behavioral markers, and suicide-related outcomes; and (2) propose a clinically interpretable structure for suicide-specific digital phenotyping to develop actionable digital markers.

## Methods

### Overview

This scoping review was conducted based on Arksey and O’Malley’s methodological framework and the PRISMA-ScR (Preferred Reporting Items for Systematic Reviews and Meta-Analyses extension for Scoping Reviews) guidelines (Checklist 1) [17,18]. The review was guided by the population-concept-context framework (population: not restricted; concept: passively collected digital data from smartphones or wearable devices; and context: suicide-related outcomes). The study was conducted through collaboration among 3 research teams, with external validation performed by separate research teams. Fourteen studies were analyzed based on the LHSF using a structured data charting and synthesis approach [16], and Google Sheets was used as a data management tool. The protocol was registered in the Open Science Framework [19].

### Search Strategy

Textbox 1 summarizes the query terms used in the search strategy. The search strategy was developed iteratively using the population-concept-context framework to ensure alignment with the review objectives. Four databases (PubMed, CINAHL, PsycINFO, and IEEE Xplore) were searched for studies published between January 1, 2015, and December 31, 2025. The search combined suicide-related terms with those related to digital sensing, as summarized in Textbox 1 (see full search strings in Multimedia Appendix 1). The final search was conducted in December 2025. Gray literature was excluded from the scope of this review. This boundary was established because the primary objective was to map granular digital signals onto the structured LHSF framework, which requires fully detailed and finalized descriptions of raw sensor processing, feature extraction, and statistical verification—details that are often omitted or partially reported in non–peer-reviewed literature. The reference list screening was conducted to identify additional relevant studies. A librarian at the affiliated medical library was consulted to review and refine the search strategy to enhance comprehensiveness and accuracy.

Textbox 1. **Summary of the search terms.**

Suicide-related terms include the following:

- Suicide
- Suicidal ideation
- Suicide attempt
- Suicide risk
- Suicidal behavior

Digital sensing terms include the following:

- Smartphone
- Wearable devices
- Passive sensing
- Digital marker
- Digital phenotype

### Eligibility Criteria

We included studies that reported correlational or predictive analyses that explored digital markers with suicide-related outcomes. Studies that relied solely on active data collection methods or did not assess suicide-related outcomes were excluded. No language restrictions were applied. Textbox 2 summarizes the detailed inclusion and exclusion criteria. The publication period was restricted to studies from January 2015 onward, corresponding to the introduction of digital phenotyping in clinical research and wearable devices [20].

Textbox 2. **Inclusion and exclusion criteria.**

Inclusion criteria

- Full-text, peer-reviewed studies (including articles published online ahead of print)
- Studies that used passively collected digital data from smartphones or wearable devices
- Studies that measured suicide-related outcomes, including suicidal ideation, plans, attempts, behaviors, or suicide risk
- Studies that analyzed associations between passive digital data and suicide-related outcomes or developed predictive models to examine suicide-related outcomes

Exclusion criteria

- Studies that used only active data collection methods (eg, online surveys, self-report questionnaires, or ecological momentary assessment apps)
- Studies that did not assess suicide-related outcomes and only focused on general mental health indicators (eg, depression, stress, or nonsuicidal self-injury)
- Feasibility studies that did not examine associations or predictive relationships between passive digital data and suicide-related outcomes
- Reviews, protocols, commentaries, conference abstracts, or non–peer-reviewed studies

### Study Selection

Two authors (SK and JK) independently screened the titles and abstracts of potentially eligible studies, followed by full-text review. If disagreements arose, they were resolved through discussion between 2 reviewers, and those not resolved were adjudicated by the principal investigator (HK).

### Data Charting

Two authors (SK and JK) initially extracted the data, which were reviewed by the principal investigator (HK). Subsequently, 2 separate teams (WTC and YZ; and TTL and ZYAT) provided external validation and contributed to refining the analysis. Discrepancies were resolved through team discussion. Google Sheets was used to organize a structured data matrix for data charting. The data charting form was developed by the research team and refined through discussion prior to full data extraction. Study characteristics, device and sensor types, and low-level features and high-level behavioral markers were charted. Narrative mapping was conducted according to the LHSF [16]. This scoping review followed Joanna Briggs Institute recommendations for scoping reviews [21]. Consistent with these guidelines, methodological quality assessment was not performed.

### Data Synthesis Using the LHSF

The LHSF was applied to organize data from raw sensor inputs to inferred behavioral patterns related to suicidality [16]. On the basis of the 4 hierarchical layers, the extracted features were grouped:

1. Sensor: hardware- or software-based sources within smartphones, wearables, or apps that passively collected environmental or behavioral data (eg, accelerometer, photoplethysmography, and phone usage logs);
2. Low-level features: quantitative indicators derived directly from raw sensor data (eg, step count and HRV);
3. High-level behavioral markers: psychosocial or behavioral patterns inferred from combinations of low-level features (eg, sleep disturbance and social withdrawal); and
4. Suicide-related outcomes: clinical indicators, such as suicidal ideation, suicide attempts, or suicide risk.

To synthesize findings across the 4 levels, we organized and categorized extracted features according to the LHSF structure. While the original framework was retained, specific digital features and behavioral markers were refined and expanded based on the included studies to reflect recent advances in digital phenotyping research. This approach enabled us to map contemporary empirical evidence onto the existing framework.

To ensure consistent operationalization across layers, explicit decision rules were applied during data charting. A feature was classified as a low-level indicator (layer 2) if it represented a directly quantifiable, sensor-derived measurement requiring no inferential interpretation. A construct was classified as a high-level behavioral marker (layer 3) if it represented a psychosocial or clinical construct that required aggregating or theoretically interpreting one or more low-level features. When the original study explicitly used a term corresponding to a layer 3 construct, it was retained as source defined. When no such label was present, 2 authors (SK and JK) independently derived a conceptually equivalent label based on the analytical context of the original study, indicated by superscript “a” in Table 1. Discrepancies in layer assignment or label derivation were resolved through discussion. A detailed overview of the operationalization criteria applied to each study is provided in Multimedia Appendix 2.

**Table 1.** Passive data sources and extracted features mapped to the layered hierarchical sensemaking framework (LHSF).

Study	Device	LHSF layer 1: sensor	LHSF layer 2: low-level features	LHSF layer 3: high-level behavioral markers	LHSF layer 4: suicide-related outcome (measures)
[22]	Smartphone	SMS text	Linguistic inquiry and word count–based word proportions (eg, first-person pronouns, emotion-related words) and communication activity (eg, message counts and sent-to-received message ratios)	Psychological and behavioral patterns^a^ (self-focus, sentiment, and social engagement)	Suicide attempts: high risk; suicidal ideation: moderate risk; depressed and positive episodes: low/minimal risk (retrospective episode-based interview)
[23]	Smartphone	Screen time app	Smartphone screen time per app category	Smartphone use patterns^a^	Suicide risk: past suicide attempts, suicidal ideation frequency, suicide threat, and perceived likelihood of death by suicide (Suicidal Behaviors Questionnaire-revised)
[24]	Wearable (wristband)	Photoplethysmography sensor	High-frequency HRV^b^ power	Reduced parasympathetic activity	Suicide severity: suicidal ideation and behavior (Columbia-Suicide Severity Rating Scale)
[25]	Smartphone, wearable (wristband)	Electrodermal activity sensor	Number of autonomic events	Autonomic arousal	Suicidal ideation (EMA^c^)
[26]	Smartphone, wearable (wristband)	Not stated but sensors embedded in wristband	Sleep minutes and step counts	Sleep patterns and activity patterns	Suicidal ideation (Patient Health Questionnaire-item 9)
[27]	Smartphone	SMS text	Incidence of sleep-related text messages, the number of nightly texts sent, and the number of unique nightly hour bins	Sleep disturbance	Suicidal ideation; suicide attempts (clinical interview)
[28]	Smartphone, wearable (wristband)	Not stated but sensors embedded in wristband	Root-mean-square of successive differences, resting heart rate, total steps, and sleep duration	Distress, physical activity, and sleep disturbance	Next-day suicidal ideation (EMA using Columbia-Suicide Severity Rating Scale-based items)
[29]	Wearable (compression shirt)	Electrocardiogram and pressure sensors	HRV indices and respiratory rate	Autonomic imbalance	Current suicidal ideation; history of suicide attempts (Schedule for Affective Disorders and Schizophrenia-Lifetime version–based clinical interview)
[30]	Smartphone	Global positioning system, actigraphy (sensor was not stated); health data aggregation app; and phone usage logs (app usage logs)	Distance walked, time spent at home, steps taken, and app usage (binary)	Behavioral changes in daily activity profiles	1-week suicide-risk events (electronic health records)
[31]	Smartphone, wearable (wristband)	Three-axis accelerometer	Time in bed, sleep onset latency, sleep duration, wake after sleep onset, and sleep efficiency	Sleep disturbance	Next-day suicidal ideation (EMA)
[32]	Smartphone	GPS	Time at home, entropy (location variability), and distance traveled	Social connectedness, behavioral withdrawal, and anhedonia	Next-week suicidal events: attempts, psychiatric hospitalization, suicide-related emergency department visits (Self-injurious Thoughts and Behaviors Interview, weekly experience sampling method, Columbia-Suicide Severity Rating Scale); same-week/next-week suicidal ideation (weekly experience sampling method)
[33]	Wearable (wristband)	Photoplethysmography sensor, accelerometer, and gyroscope	Heart rate and HRV, movement index, wear-free time, step count, walking/running time, other exercise time, resting time, and total sleep time	Stress states (autonomic dysregulation), daily behavioral patterns^a^	Elevated suicide risk: suicidal ideation and behavior (Hamilton Depression Rating Scale-item 3)
[34]	Smartphone	Screen capture app	Dictionary-based word counts	Expression related to suicide risk^a^	Passive/active suicidal ideation, suicidal planning (EMA)
[35]	Smartphone	Screen capture app	Smartphone screen time (screenshot image counts, any use, and proportion of use)	Temporal patterns of smartphone use^a^	Suicide risk: suicidal ideation, suicidal planning, and suicidal desire (EMA)

a These categories represent high-level behavioral markers (LHSF layer 3) derived by the authors and were not explicitly defined as such in the original studies.

b HRV: heart rate variability.

c EMA: ecological momentary assessment.

### Synthesis of Evidence Strength Across Feature Domains

To summarize the strength of evidence across feature domains, each study was classified according to the statistical results reported in the original article. A study was counted as showing a significant association for a given feature domain if at least one indicator in that domain was significantly associated with, or predictive of, at least one suicide-related outcome, such as suicidal ideation, planning, attempts, risk, or events. This classification was retained even when other indicators or outcomes within the same study were nonsignificant. Domain-level evidence was then described by the number of studies reporting significant associations, rather than by a single subjective judgment. Predictive performance was interpreted against a conventional discrimination threshold of an area under the curve (AUC) of 0.80 or above.

## Results

### Selection of Sources of Evidence

**Figure 1.**
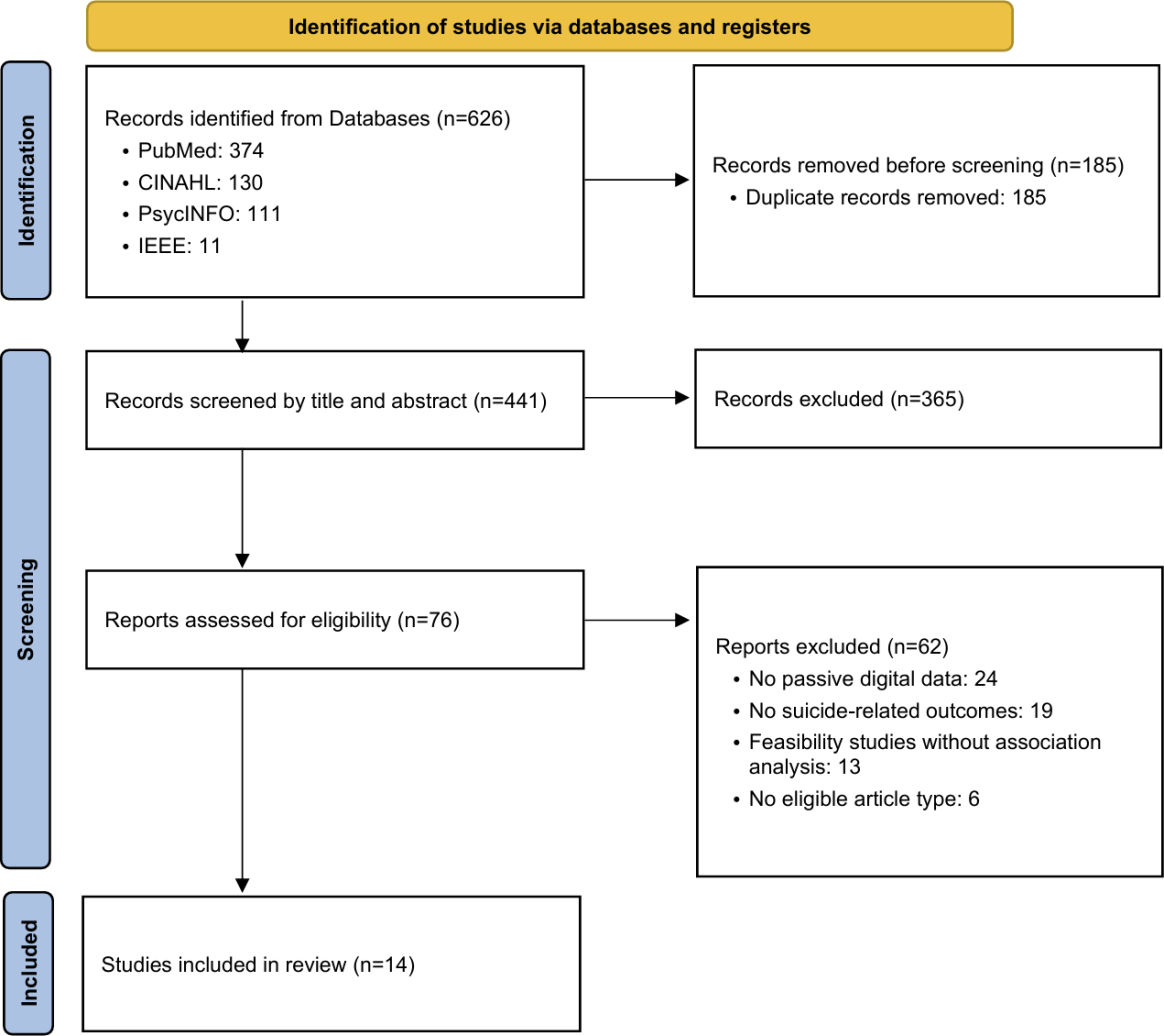
PRISMA (Preferred Reporting Items for Systematic Reviews and Meta-Analyses) flow diagram.

We conducted the final search in December 2025 and identified 626 studies across the 4 databases (PubMed: n=374; CINAHL: n=130; PsycINFO: n=111; and IEEE Xplore: n=11). After removing 185 duplicate records, 441 (70.4%) articles were screened based on their titles and abstracts. Of these, 365 (82.8%) records were excluded, and 76 (17.2%) full-text articles were assessed. Subsequently, 62 (81.6%) articles were excluded (Figure 1). Ultimately, 14 (18.4%) studies were included [22-35]. Reference list screening identified no additional eligible articles.

### Characteristics of the Included Studies

Table 2 presents the characteristics of the included studies. Publication years of the 14 included studies ranged from 2020 to 2025, with most of the studies published in 2025 (n=5, 35.7%). Most studies were conducted in the United States (n=11, 78.6%). All included studies used observational designs, in which 12 (85.7%) were longitudinal studies, and 2 (14.3%) were cross-sectional. Furthermore, 11 (78.6%) studies were in prospective design, and 3 (21.4%) were retrospective ones. Regarding characteristics of participants, 3 (21.4%) studies included adolescents, and 11 (78.6%) studies included only adults. Most studies targeted high-risk populations (n=12, 85.7%), such as individuals who visited emergency departments for suicide-related reasons, psychiatric inpatients or outpatients, and those with a history of suicidal ideation or behavior. Duration of passive data collection varied across studies, ranging from 24 hours to 6 months.

**Table 2.** Characteristics of the included studies (N=14).

Author (year)	Country	Study design	Study participants			Passive data collection period
			Size, n	Types	Characteristics (female ratio/setting)	
Glenn et al (2020) [22]	United States	Observational Longitudinal Quantitative Retrospective Pilot study	33	High-risk adults who reported a past suicide attempt Age: mean 20.4 (SD 2.4) y	Female ratio: 84.8% Community setting	2 wk
Coyne et al (2021) [23]	United States	Observational Cross-sectional^a^ Quantitative Prospective (cohort)	281	General adolescents and emerging adults Age: mean 23.3 (SD 1.04) y	Female ratio: 58% Community setting	2 wk
Sheridan et al (2021) [24]	United States	Observational Longitudinal Quantitative Prospective	51	High-risk adolescents Age: mean 16.7 (SD 1.5; range 14-21) y	Female ratio: 72.6% Emergency department or psychiatric inpatient unit	7 d
Kleiman et al (2021) [25]	United States	Observational Longitudinal Quantitative Prospective (model fit improvement)	25	High-risk adults Age: mean 33.48 (SD 13.84, range 19-63) y	Female ratio: 56% Psychiatric inpatient unit	Inpatient stay +28 d after discharge
Horwitz et al (2022) [26]	United States	Observational Longitudinal Quantitative Prospective (cohort)	2881	General adults (medical interns) Age: mean 27.6 (SD 2.6) y	Female ratio: 56.8% Community setting	2‐3 mo
Ladis et al (2023) [27]	United States	Observational Longitudinal Quantitative Retrospective Pilot study	26	High-risk young adults who reported past suicide attempts Age: mean 20.8 (SD 2.6) y	Female ratio: 84.6% Community setting	2 wk
Czyz et al (2023) [28]	United States	Observational Longitudinal Quantitative Prospective	102	High-risk young adults Age: mean 20.9 (SD 2.1, range 18-25) y	Female ratio: 81.4% Emergency department	8 wk
Ortiz et al (2024) [29]	Canada	Observational Cross-sectional Quantitative Retrospective Pilot study	53	High-risk adults Age: mean 44.7 (SD 13.1) y	Female ratio: 66% Psychiatric outpatient clinics	24 h
Barrigon et al (2023) [30]	Spain	Observational Longitudinal Quantitative Prospective	225	High-risk adults Age: mean 43.24 (SD 14.13 y)	Female ratio: 62.7% Psychiatric outpatient clinic	6 mo
Mournet et al (2025) [31]	United States	Observational Longitudinal Quantitative Prospective Case series	7	High-risk adults Age: mean 28 (SD 10.39, range 19-50) y	Female ratio: 71.4% Psychiatric outpatient program	6 wk
Auerbach et al (2025) [32]	United States	Observational Longitudinal Quantitative Prospective Case series	186	High-risk adolescents Age: mean 16.43 (SD 1.68, range 13-18) y	Female ratio: 79.6% Psychiatric outpatient programs, emergency departments, or community-based recruitment	6 mo
Um et al (2025) [33]	Republic of Korea	Observational Longitudinal Quantitative Prospective	59 (patient group: n=39; healthy controls: n=20)	High-risk adults and healthy controls Age range 20-55 y	Female ratio: 57.6% Psychiatric outpatient clinic; healthy controls recruited via advertisement	2 mo
Ammerman et al^b^ (2025) [34]	United States	Observational Longitudinal Quantitative Prospective	79	High-risk adults who reported past-month active suicidal ideation or behaviors Age: mean 35.15 (SD 11.07; range: 20‐63) y	Female ratio: 68.3% Community setting	28 d
Jacobucci et al^b^ (2025) [35]	United States	Observational Longitudinal Quantitative Prospective	79	High-risk adults who reported past-month suicidal ideation or behaviors Age: mean 35.15 (SD 11.07; range: 20‐63) y	Female ratio: 68.3% Community setting	28 d

a Passive data were collected and analyzed cross-sectionally over a 2-week period within a larger longitudinal project.

b Studies 34 and 35 utilized the same dataset; however, they analyzed different independent variables (passive sensing-based text patterns vs. smartphone screen time).

Regarding demographic composition, most study samples were predominantly White or Caucasian, with White participants comprising 57% to 90% of samples across studies reporting race or ethnicity. Female participants comprised the majority across all studies, with female ratios ranging from 56% to 84.8%. Although most studies recruited high-risk adult clinical populations, 3 (21.4%) studies included adolescents. Among studies reporting clinical diagnoses, depressive and anxiety disorders were the most represented conditions. Socioeconomic status was inconsistently reported; where reported, samples tended to include participants with different levels of college education or current employment. Detailed demographic and clinical sample characteristics are presented in Multimedia Appendix 3.

### Passive Data Sources and Extracted Features Mapped to the LHSF

#### Overview

We mapped the passive data sources and features extracted from the 14 studies according to the 4-layer structure of the LHSF (Table 1) [16]. Results were presented from data sources to suicide-related outcomes. High-level behavioral markers (layer 3) were labeled using the terminology reported in the original studies. When not explicitly defined, conceptually similar descriptors derived by the authors were indicated with the superscript “a” (Table 1).

#### LHSF Layer 1: Sensors

Passive sensing devices were categorized as smartphones and wearable devices. All wearable devices were wristband based, except for 1 study that used a compression shirt equipped with sensors to measure the respiratory rate [29]. Smartphones were used either alone or in combination with wearable devices in studies that assessed suicide-related outcomes via EMA [25,28,31,34,35] or experience sampling method [32].

Wearable devices primarily collected physiological signals, including photoplethysmography [24,33], electrodermal activity [25], accelerometer and gyroscope data [33], electrocardiogram signals [29], and pressure sensor data [29], which were used to derive indicators, such as HRV and autonomic nervous system responses. In contrast, smartphones captured behavioral data, including location information via a global positioning system [30,32], accelerometer-based sleep metrics [31], screen time [23], screen capture data [34,35], phone usage logs [30], and SMS text data [22,27].

#### LHSF Layer 2: Low-Level Features

Low-level features were extracted from sensor data and categorized into physiological, behavioral, and digital indicators. Physiological indicators included heart rate and HRV indices, respiratory rate, and electrodermal activity [24,25,28,29,33]. Behavioral and physical activity indicators comprised step counts, distance traveled, exercise and rest duration, total sleep time, and mobility entropy [26,28,30-33]. Digital behavioral indicators included smartphone screen time and phone usage logs [23,30,35], dictionary-based word counts [34], number of nighttime text messages sent [27], and ratios of sent-to-received text messages [22]. Studies that analyzed text message content included dictionary-based linguistic features, such as emotion-related or sleep-related word usage [22,27].

#### LHSF Layer 3: High-Level Behavioral Markers

High-level behavioral markers were derived through an integration of low-level features. Studies (n=4, 28.6%) that leveraged physiological low-level features mapped these indicators to markers that reflected reduced parasympathetic activity or autonomic dysregulation, corresponding to altered autonomic nervous system regulation [24,25,29,33].

Studies (n=6, 42.9%) based on behavioral low-level features identified high-level markers that captured changes in overall daily activity profiles. These markers included sleep patterns or sleep disturbance, changes in activity patterns and activity levels, social connectedness, behavioral withdrawal, and anhedonia, which reflected behavioral and psychosocial characteristics [26,28,30-33].

Studies (n=4, 28.6%) that used smartphone-based digital behavioral features and reported high-level markers included smartphone use patterns or temporal changes in use, changes in daily activity profiles, and sleep disturbance [23,27,30,35]. In addition, 3 (21.4%) studies derived high-level markers from text-based analyses, which included expressions related to suicide risk, self-focus, sentiment, and social engagement, representing psychological and behavioral patterns [22,27,34].

#### LHSF Layer 4: Suicide-Related Outcomes

Suicide-related outcomes were assessed using clinical measures and real-time monitoring. Outcomes included suicidal ideation and planning, suicide attempts, severity, risk, and events.

Many studies reported suicide-related outcomes measured via EMA or experience sampling methods [25,28,31,32,34,35], including those that administered standardized clinical questionnaires via EMA [28]. In addition, several studies assessed suicide-related outcomes via structured clinical interviews or standardized questionnaire items [23,24,26,28,29,32,33]. The Columbia-Suicide Severity Rating Scale was the most frequently used clinical instrument [24,28,32]. One study identified suicide-related events from electronic health records [30].

### Associations and Predictive Relationships Between Passive Digital Markers and Suicide-Related Outcomes

#### Overview

Table 3 presents the statistical analyses and main findings of the relationships between passively collected digital features and suicide-related outcomes across the 14 studies.

**Table 3.** Association and prediction between passive digital markers and suicide-related outcomes.

Study	Outcome type	Main statistical analysis methods	Predictive metric	Predictive performance	Main findings	Further suggestion
[22]	Association	Mixed effects models (generalized linear and linear mixed effects models)	Not applicable	Not applicable	Steeper increases in anger (*χ*²_3_=7.83; *P*=.049) and steeper decreases in positive emotion (*χ*²_3_=41.67, *P*<.001) over time were associated with suicide attempt episodes (high risk) compared with other episodes (moderate risk, minimal or no risk). Increases in self-focus did not uniquely distinguish suicide attempt episodes from all other episode types (*P*=.37 when compared to positive mood episodes).	Prospective, frequently assessed designs Objective classification of suicide-related events Larger samples for adequate power Development of machine learning models for real-time prediction of suicide risk Advanced Natural Language Processing beyond dictionary-based methods
[23]	Association	Zero-inflated negative binomial regression and hierarchical regression analysis	Not applicable	Not applicable	Higher use of entertainment apps in girls and reading apps in boys was associated with increased suicide risk (girls: β=.23‐.25; *P*<.001; boys: β=.28‐.29; *P*<.001). Video game use was associated with higher suicide risk among boys experiencing cybervictimization (β=.27; *P*=.01).	Replication in clinical high-risk samples Longitudinal studies incorporating passive sensing from earlier waves Repeated baseline and longitudinal assessment of suicide risk Examination of contextual factors associated with media use
[24]	Association	Mixed effects models (random effect for patient)	Not applicable	Not applicable	Increased high-frequency HRV^a^ at night was significantly associated with decreased suicide severity (mean difference=11.89 ms/√Hz; *P*=.005).	Validation in larger cohorts Improvement of wearable data quality Inclusion of additional HRV metrics
[25]	Prediction (model fit improvement)	Linear and generalized linear mixed effects models	Performance score	Performance score improvement (hourly level): mean 14.2% (2.7%‐22.9%) Performance score improvement (daily level): mean 18.1% (range: 5.4%‐31.9%)	Electrodermal activity–based autonomic events improved model performance for predicting suicidal ideation severity (performance score improvement up to 31.90%) but worsened model fit for the presence of suicidal ideation.	Replication in larger samples Algorithm refinement to distinguish psychological distress from nonspecific physiological activation
[26]	Prediction	Three-step hierarchical logistic regression	AUC^b^	AUC: 0.74	Passively collected sleep and physical activity features had no incremental predictive value for suicidal ideation and only negligible predictive value for depression (suicidal ideation: *χ*²_4_=0.7; *P*=.95; depression: *χ*²_4_=13.5, *P*=.009).	Refinement of passive sensing features Optimization of prediction windows and data processing strategies Development of adaptive interventions based on early risk indicators
[27]	Association	Generalized linear mixed models, negative binomial models, and multilevel ordinal logistic regression	Not applicable	Not applicable	Sleep-related linguistic features were not significantly associated with suicidal ideation or suicide attempt episodes (suicidal ideation: z=1.05; *P*=.29; and suicide attempt: z=1.88; *P*=.06), and nocturnal texting patterns were also not significantly associated with these outcomes (suicidal ideation: z=0.61; *P*=.54; and suicide attempt: z=0.83, *P*=.41).	Integration of wearable sensors for objective sleep assessment Refinement and validation of sleep-related linguistic dictionaries Replication in larger and more diverse samples
[28]	Prediction	Multilevel machine learning (based classification and regression tree models)	AUC	AUC (ecological momentary assessment only): 0.84 AUC (passive only): 0.56 AUC (ecological momentary assessment+ passive combined): 0.84	Self-reported ecological momentary assessment features had excellent accuracy in predicting next-day suicidal ideation. Passive sensor data alone had poor predictive performance and did not improve the model when combined.	Prediction of suicide attempts beyond ideation Replication in independent samples for external validation Application of alternative machine learning approaches Optimization of assessment frequency to balance burden and accuracy
[29]	Association	Logistic regression	Not applicable	Not applicable	Suicide attempt history was associated with increased respiratory rate (*U*=14.1; *P*<.01) and higher low-frequency to high-frequency HRV ratio (*Wald χ*²=4.1; *P*=.04). Current suicidal ideation was associated only with a higher low-frequency to high-frequency HRV ratio (*Wald χ*²=3.8; *P*<.05).	Longitudinal studies on diaphragmatic breathing Mechanistic research on respiratory rate and hypocapnia
[30]	Prediction	Machine learning (unsupervised model and Bayesian change-point detection)	AUC	AUC: 0.78	Behavioral changes detected from passive smartphone data predicted 1-wk suicide risk events, including suicide attempts and psychiatric emergency visits.	Integrate passively collected smartphone data with actively collected self-report data, electronic health records, and clinical assessment data Include a broader range of passively collected behavioral and environmental data to enhance short-term suicide risk prediction
[31]	Association	Individual linear regressions	Not applicable	Not applicable	Among actigraphy-derived sleep metrics (time in bed, sleep onset, sleep duration, sleep efficiency, and wake after sleep onset), only wake after sleep onset had a significant within-person association with next-day suicidal ideation in one of 7 participants (*b*=0.04; *P*=.02).	Replicate in larger and more diverse samples Examination of sleep regularity metrics Individual-level analysis of actigraphy data
[32]	Prediction^c^	Repeated-measures mixed effects logistic regression models	AUC	AUC: 0.64	Greater within-person increases in homestay were related to next-week suicidal events (aOR=1.99; *P*=.01). Entropy and distance traveled were not (entropy: *P*=.14; distance: *P*=.13), and geolocation features were not related to next-week suicidal ideation (homestay: *P*=.90; entropy: *P*=.56; and distance: *P*=.38).	Multimodal sensor and clinical data integration External validation to improve generalizability
[33]	Prediction	Machine learning models	AUC	AUC (single-step model): 0.88 AUC (multistep model): 0.89	HRV-related features and clinician-rated depression severity were key predictors of an elevated suicide risk (Hamilton Depression Rating Scale-item 3≥1) in patients with depressive disorders. Activity and sleep data had limited predictive value.	Refining temporal resolution for real-time tracking Expanding digital phenotypes (eg, geolocation, communication patterns) Increasing sample size and diversity Improving wearable adherence External validation of machine learning models
[34]	Association	Zero-inflated negative binomial regression, Spearman correlation	Not applicable	Not applicable	Passively collected smartphone text patterns related to suicide risk were significantly associated with passive and active suicidal ideation (*rₛ*=0.05‐0.07, *P*<.05) and with suicidal planning based on within-person zero-inflated negative binomial effects (*P*<.05).	Examine specific words driving divergent dictionary effects Account for cooccurrence of suicidal ideation and planning Apply flexible natural language processing or machine learning methods
[35]	Association	Bayesian zero-inflated negative binomial multilevel model	Not applicable	Not applicable	Within-person increases in smartphone screen time were associated with higher near-term (<3 h) suicidal ideation (passive: *b*=0.04, 95% CI 0.02‐0.06; active: *b*=0.04, 95% CI 0.02‐0.07) and suicidal planning (*b*=0.15, 95% CI 0.02‐0.26).	Examination of screen content and contextual factors Refinement of temporal modeling Validation in larger and more diverse samples

a HRV: heart rate variability.

b AUC: area under the curve.

c Ref 32 reported both associational (effect estimates) and predictive (AUC) findings from a single modeling approach and was classified as a predictive study for the analytic-approach count.

#### Statistical Analyses and Predictive Modeling

Across the 14 studies, 6 (42.9%) used predictive modeling approaches [25,26,28,30,32,33], and 8 (57.1%) conducted association or correlational analyses [22-24,27,29,31,34,35]. One predictive study [32] also reported association findings from the same modeling framework and was therefore included in the predictive group. Among studies that used predictive models, reported model performance varied, and AUC values ranged from 0.56 to 0.89.

#### Physiological Indicators

All 4 (28.6%) studies examining physiological digital features related to autonomic regulation reported at least one significant association with suicide-related outcomes [24,25,29,33]. Increased high-frequency HRV at night was significantly associated with decreased suicide severity [24]. Autonomic arousal events derived from electrodermal activity improved the model performance for predicting the severity of suicidal ideation; however, adding these features worsened model fit when the outcome was the presence of suicidal ideation [25]. Lower HRV-related features were identified as key predictors of an elevated suicide risk with excellent discrimination (AUC=0.89 for the multistep model) [33]. In contrast, physiological activity and sleep data had limited predictive value for suicide risk [33]. Individuals with a history of suicide attempts also exhibited increased respiratory rates and higher low-frequency to high-frequency HRV ratios compared with those without such history [29].

#### Mobility and Geolocation Indicators

Two (14.3%) studies examining mobility and geolocation-derived behavioral markers reported at least one significant association with suicide-related outcomes [30,32]. Behavioral changes derived from smartphone data predicted suicide risk events over 1 week with acceptable classification performance (AUC=0.78) [30]. In one predictive study that also examined within-person associations [32], greater increases in homestay were significantly associated with next-week suicidal events (adjusted odds ratio=1.99, *P*=.01); however, predictive accuracy based on the homestay model was modest (AUC=0.64). Other geolocation features, such as entropy and distance traveled, were not significant predictors of suicidal incidents. Additionally, no geographical information was associated with next-week suicidal ideation [32].

#### Smartphone Use Patterns

Two (14.3%) studies examining smartphone use patterns reported significant associations with suicide-related outcomes [23,35]. Higher entertainment and reading app use in girls and boys, respectively, were associated with increased suicide risk; additionally, video game use was associated with elevated risk among boys who had experienced cybervictimization [23]. Within-person increases in smartphone screen time were associated with higher levels of near-term (within 3 h prior to EMA assessment) suicidal ideation and planning [35].

#### Sleep Indicators

One case series study examined participant-specific associations between actigraphy-derived sleep features and next-day suicidal ideation using individual-level (within-person) regressions [31]. Assessed sleep metrics included time in bed, sleep onset, sleep duration, sleep efficiency, and wake after sleep onset. Among the 5 sleep metrics assessed, only wake after sleep onset was significantly associated with higher next-day suicidal ideation, and this association was observed in only 1 of the 7 participants. Sleep features examined in predictive contexts likewise showed no incremental predictive value [26,28].

#### Linguistic and Communication Markers

Three studies examined linguistic and communication-based markers derived from smartphone text data [22,27,34]. Of these, 2 reported at least one significant association [22,34], and 1 reported no significant association [27]. In a study by Ammerman et al [34], smartphone text patterns related to suicide risk were significantly associated with both passive and active suicidal ideation, as well as suicidal planning. In a retrospective episode-based analysis [22], trajectories of emotion-related language differed across suicide risk levels: before suicide attempts, anger increased and positive emotion decreased over 2 weeks, although self-focus did not consistently differentiate episode types. In contrast, sleep-related linguistic features and nocturnal texting patterns were not significantly associated with suicidal ideation or suicide attempts [27].

### Nonsignificant or Limited Predictive Performance

Two (14.3%) studies reported nonsignificant or limited predictive performance for sleep- and activity-related passive features [26,28]. In predictive contexts, passively collected sleep and physical activity features had limited or no incremental predictive value for suicidal ideation when compared with, or added to, clinician-rated or self-reported measures [26,28]. In one study, passive sensor data alone demonstrated poor predictive performance for next-day suicidal ideation (AUC=0.56) and did not improve prediction when combined with EMA features [28].

### Future Research Directions Identified Across Studies

Across the included studies, several directions for future research were identified (Table 3). First, many studies emphasized the need for replication in larger and more diverse samples, along with the use of longitudinal designs, and more frequent assessments to improve the robustness and generalizability of findings [22-24,27,28,31,33,35]. Second, a consistent focus was placed on improving data quality and methodological approaches, including refinement of passive sensing features, optimization of temporal resolution, and the application of more advanced analytic techniques such as machine learning and natural language processing [22,25-28,33-35]. Third, these findings collectively highlight the critical need to bridge the gap between passively collected digital data and clinically interpretable indicators of suicide risk [30,32]. Although diverse data streams and analytic approaches are increasingly used, there remains a lack of structured approaches to translate low-level digital features into meaningful behavioral or clinical constructs. This underscores the importance of developing frameworks that can systematically organize and represent the relationships between raw data and clinically relevant suicide-related outcomes. Further details are provided in Table 3.

### LHSF-Based Conceptual Synthesis of Passive Digital Markers and Suicide-Related Outcomes

**Figure 2.**
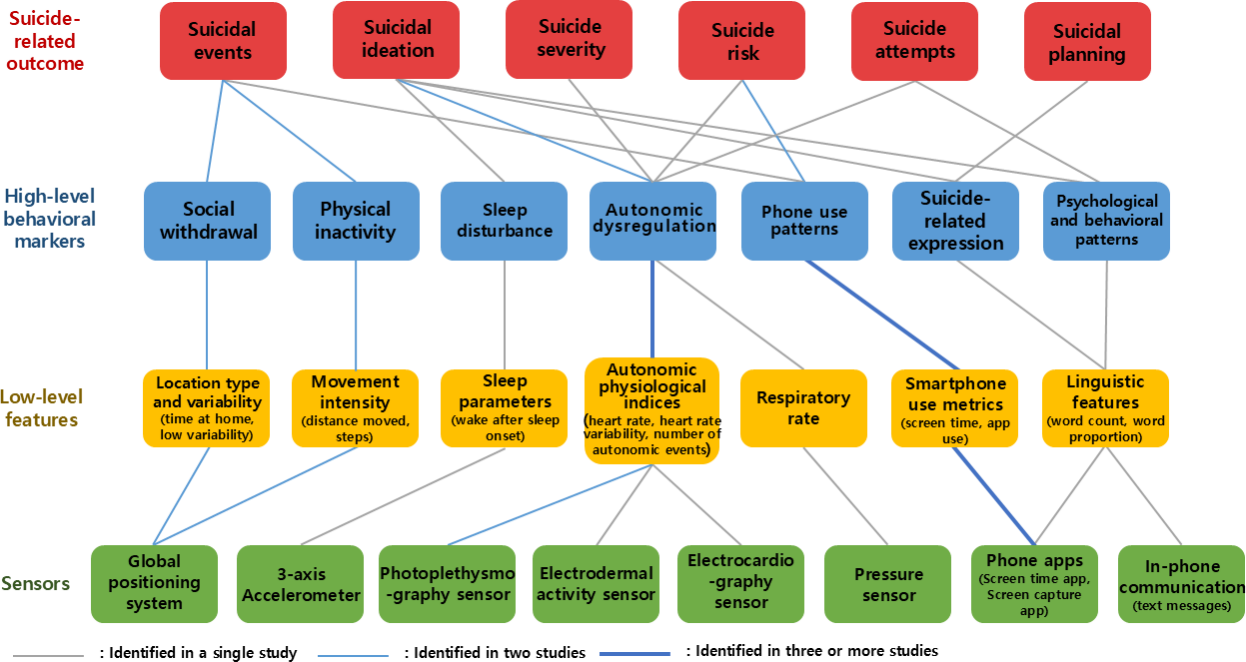
Layered hierarchical sensemaking framework–based conceptual mapping of reported associations between passive digital data and suicide-related outcomes.

Figure 2 presents the LHSF-based conceptual mapping synthesizing the reported associations between passive digital markers and suicide-related outcomes. Low-level features extracted from passive sensors consisted of 7 domains, including location type and variability, movement intensity, sleep parameters, autonomic physiological indices, respiratory rate, smartphone use metrics, and linguistic features.

Across the 4 layers of the LHSF, physiological sensor-derived features were linked to autonomic dysregulation markers in all 4 relevant studies and showed associations with very diverse outcomes, including suicidal ideation, suicide severity, risk, and attempts. Behavioral and mobility-related features derived from the global positioning system and accelerometers mapped to high-level markers that captured social withdrawal, physical inactivity, and sleep disturbance. These markers showed associations with suicidal ideation and suicide-related events. Smartphone use metrics mapped to smartphone use patterns and were linked to suicide-related events and risk indicators, including suicidal ideation and planning. Linguistic features extracted from text messages mapped to high-level markers that reflected expression related to suicide risk and psychological and behavioral patterns. These markers were associated with suicidal ideation, planning, and suicide attempts. Overall, mapping revealed that passive digital markers were represented across multiple hierarchical levels of the LHSF and linked to diverse suicide-related outcomes.

## Discussion

### Main Findings

This scoping review proposed a suicide-specific adaptation of the LHSF to integrate fragmented evidence from digital sensing studies into clinically meaningful behavioral markers in suicide research. The adapted LHSF organized diverse digital features into higher-order constructs, such as autonomic dysregulation, sleep disturbance, and psychosocial dysfunction, and provided a conceptual bridge between technical sensor outputs and clinically interpretable suicide-related states.

### Physiological Indicators

Autonomic indicators, particularly HRV-related features, were significantly associated with suicide-related outcomes in all 4 relevant studies and achieved the highest predictive performance among the included studies (AUC=0.89) [33]. Beyond prediction, HRV and autonomic measures were repeatedly associated across the broadest range of suicide-related outcomes in correlational studies, including suicide severity, current suicidal ideation, and attempt history [24,29]. However, they should be interpreted alongside other behavioral and contextual markers [24,36]. This pattern is consistent with prior evidence linking autonomic dysregulation to suicide risk [36,37]. Suicidality was associated with dysregulation of the autonomic nervous system, reflected in reduced parasympathetic activity and altered HRV [36]. Electrodermal activity improved model performance for predicting the severity of suicidal ideation; however, it worsened model fit when the outcome was the presence of suicidal ideation [25].

Passive physiological monitoring, including HRV assessment using wearable devices, might complement conventional suicide risk assessments, particularly for individuals who did not accurately disclose their suicidal ideation or behavior. Passively collected physiological data could be processed into HRV indices that reflect autonomic nervous system regulation and dysregulation [38,39]. These physiological changes could be clinically meaningful, as substantial alterations in HRV might be interpreted as indicators of psychological distress. However, as predictive performance varied across studies and outcomes, such measures should be appropriately considered as objective correlates associated with suicide-related risk rather than stand-alone clinical markers.

### Mobility and Geolocation Indicators

Mobility- and geolocation-derived indicators suggest that changes in spatial behavior may reflect proximal signals of suicide-related risk, particularly when considered as within-person deviations rather than absolute behavioral levels. Increases in homestay and reductions in mobility were associated with subsequent suicidal events, indicating that disruptions in daily movement patterns might capture short-term risk dynamics [30,32]. These behavioral shifts might represent early manifestations of psychosocial withdrawal and loss of daily structure, both well-established precursors of suicidal crises, rather than reflect isolated symptoms. Therefore, abrupt deviations from an individual’s routine can be interpreted as signals of functional deterioration difficult to capture using conventional self-reporting methods.

However, the predictive value of geolocation features differed across indicators; measures such as entropy and distance traveled were not significantly associated with suicide outcomes [32]. These findings could suggest that mobility-based markers might be sensitive to contextual and individual variability and thus should be interpreted in relation to personalized baseline patterns. From a clinical perspective, this supports an approach that prioritizes within-person changes over static thresholds, reflecting a broader shift toward dynamic and temporally sensitive models for suicide risk assessment [28].

### Smartphone Use Patterns

Although smartphone use patterns captured dynamic behavioral changes associated with short-term suicide-related outcomes, their interpretation remained highly context dependent. When individuals spend more time on their smartphones, they are more likely to experience near-term suicidal ideation and planning, suggesting that heightened device engagement might reflect acute psychological states preceding suicide risk [35]. Research indicated significant associations between specific app usage patterns and suicide risk, with certain types of media use being linked to elevated risk under specific conditions [23]. In addition, behavioral changes derived from passively collected smartphone usage data demonstrated the ability to predict suicide-related events over short time frames, including suicide attempts and psychiatric emergency visits [30].

As a result, smartphone-derived behavioral data could serve as real-time proxies for the underlying cognitive and emotional processes. However, digital behaviors were inherently ambiguous and thus might reflect multiple psychological functions, including coping, avoidance, and social interaction. Hence, smartphone-based indicators should be interpreted with caution and combined with other data sources such as EMA or clinical assessments, to improve their reliability and clinical interpretability.

### Sleep Indicators

Few sleep-related indicators were significantly associated with suicidal outcomes [31]. Only one actigraphy-derived metric, wake after sleep onset, was significantly associated with next-day suicidal ideation, and only in 1 of 7 participants at the individual level; other sleep parameters were not significantly associated [31]. A likely explanation is methodological rather than substantive. As actigraphy infers sleep from movement, it detects sleep with high sensitivity but poorly identifies wakefulness, so periods of quiet wakefulness are often misclassified as sleep, and metrics such as wake after sleep onset are often underestimated [40]. Self-reported sleep, in contrast, may capture the subjective distress more proximally linked to suicidal states, which could explain why passively sensed sleep metrics may show fewer significant associations. These findings suggest that the associations observed here may reflect current measurement constraints rather than the absence of a meaningful link. Methodological refinements, such as validating movement-based estimates against polysomnography and averaging across multiple nights to obtain more stable parameter estimates [41], together with larger samples and more consistent measurement strategies, may strengthen future findings and clarify the role of sleep-related digital markers.

### Linguistic and Communication Markers

Linguistic and communication-based indicators derived from smartphone data provided additional insights into suicide-related cognitive and emotional states, although findings varied across specific features. Text-based patterns that reflected suicide-related content were significantly associated with both passive and active suicidal ideation and planning [34]. Furthermore, longitudinal analyses demonstrated that temporal changes in emotion-related language, such as increases in anger and decreases in positive emotions, could differentiate suicide attempts from lower-risk states [22]. These findings suggest that language use may capture shifts in affective and cognitive processes preceding suicidal behavior.

However, not all linguistic features were significantly associated with suicide-related outcomes. Self-focus and sleep-related language patterns did not reliably distinguish between suicide risk states in some analyses [22,27]. Overall, linguistic markers may provide valuable indicators of internal psychological states. However, their clinical utility depends on integrating multiple features and considering variability across individuals and contexts.

### Clinical Implications

These findings highlight the potential of passive digital markers for clinically meaningful suicide risk monitoring. In practice, the continuous monitoring of physiological, behavioral, and digital signals helps expand our assessment beyond static, retrospective viewpoints toward more dynamic and temporally sensitive models of suicide risk [12]. Approaches that prioritize within-person deviations from individual baseline patterns may enable earlier detection of subtle changes preceding suicidal crises, particularly among those who may not disclose suicidal ideation or behavior [14,34].

However, the predictive performance reported across the included studies requires careful interpretation. Among the predictive studies, AUC ranged from 0.56 to 0.89. In clinical screening contexts, AUC values of 0.80 or above are generally considered the minimum threshold for adequate discrimination [42]. By this standard, only one study achieved excellent performance (AUC=0.89) [33], whereas one study approached but did not reach this threshold (AUC=0.78) [30]. The remaining studies reported AUCs below this threshold (range 0.56-0.74) [26,28,32], and one predictive study reported only relative model performance improvements rather than an AUC [25]. These findings indicate that passive digital sensing for suicide risk prediction is still at an early stage and does not yet meet the performance levels required for clinical screening tools. Nevertheless, these AUC values should be interpreted in light of the inherent difficulty of predicting rare events such as suicidal behaviors and the reliance on passive data alone without integration with clinical assessments.

From a clinical perspective, these findings suggest that passive digital monitoring systems may function as continuous and low-burden safety nets and identify emerging risks in everyday contexts. In addition, integrating these digital markers into intervention frameworks, such as Just-In-Time Adaptive Interventions, may facilitate timely and context-sensitive responses, supporting early intervention before suicidal crises intensify [43]. These approaches may be particularly useful in community and outpatient settings, where continuous monitoring using only conventional assessment methods is unfeasible. Specifically, our findings suggest that physiological markers, such as HRV, which showed the strongest predictive performance and the broadest range of associations in our review, combined with behavioral and linguistic indicators, may provide a more context-sensitive approach to suicide risk assessment.

### Study Limitations and Future Research

This review has several limitations. First, the relatively small number of included studies (n=14) limits the generalizability of our findings and the ability to establish a robust consensus on digital markers. Second, the lack of standardized data collection and extraction of suicide-related digital features across studies may have contributed to inconsistencies in the findings and limited comparability between studies. This issue is further compounded by simplified device classifications that do not account for variability across device brands, models, or operating systems (eg, iOS vs Android). Third, as a scoping review, this study did not formally assess the methodological quality of the included studies or perform a quantitative synthesis, such as a meta-analysis. Fourth, the exclusion of gray literature may have introduced publication bias. Although peer-reviewed publications were targeted to ensure complete technical descriptions for LHSF mapping, relevant negative or null results may exist outside these databases, potentially narrowing our synthesized evidence base. Fifth, most studies (78.6%) were conducted in the United States, and included samples were predominantly White or Caucasian samples and mostly female participants. This demographic and geographic homogeneity limits the generalizability of the present findings to culturally and demographically diverse populations. These limitations reflect the emerging nature of research in passive digital sensing for suicide-related outcomes and highlight the need for more rigorous and standardized methodological approaches. However, this review provides a structured framework for selecting digital markers for suicide prevention and offers a foundation for future research in digital suicide phenotyping.

Several directions for future research have emerged based on this review. First, future studies should adopt prospective longitudinal or real-time designs to examine multimodal integration, combining passive sensor data with EMA and electronic health record data to enhance clinical context and interpretability. Second, large-scale validation studies using advanced analytic approaches, such as machine learning and natural language processing, are needed, along with rigorous external validation, to improve the discrimination between general psychological distress and suicidal crises. Third, future research should move beyond correlational and predictive modeling and focus on intervention-focused research, such as clinical trials to develop and test actionable digital phenotypes within intervention frameworks, such as Just-In-Time Adaptive Interventions frameworks. These approaches may enable real-time clinical alerts or timely responses when specific digital markers are detected. Fourth, future reviews in the field of digital suicide phenotyping should consider incorporating gray literature to mitigate potential publication bias and establish a more comprehensive evidence base. Fifth, future research should prioritize the inclusion of demographically and geographically diverse samples to strengthen the generalizability and clinical translatability of passive digital markers across cultural contexts.

### Conclusions

This scoping review provides a suicide-specific adaptation of the LHSF to organize fragmented evidence on passive digital sensing into clinically interpretable behavioral markers. Physiological indicators related to autonomic regulation were associated with suicide-related outcomes across all relevant studies and showed the strongest predictive performance (AUC=0.89). Furthermore, smartphone-derived behavioral markers demonstrated additional potential for capturing proximal changes in suicidal states, whereas linguistic markers showed more variable findings. In contrast, sleep- and activity-related indicators showed few significant associations. This review clarifies how passive digital features can be translated into higher-order behavioral constructs and advances the conceptual foundation for digital suicide phenotyping. These findings highlight the potential of integrating multimodal digital data to enable continuous, real-time monitoring of suicide risk in natural settings. Future research should focus on improving the reliability, interpretability, and external validity of digital markers to develop clinically actionable systems for early detection and timely intervention in high-risk populations.

## Supplementary material

10.2196/98616 Multimedia Appendix 1 Search queries.

10.2196/98616 Multimedia Appendix 2 Operationalization criteria for assigning features to LHSF layers 2 and 3.

10.2196/98616 Multimedia Appendix 3 Demographic and clinical sample characteristics of the included studies.

10.2196/98616 Checklist 1 PRISMA-ScR checklist.

## Abbreviations

AUC: area under the curve
EMA: ecological momentary assessment
HRV: heart rate variability
LHSF: Layered Hierarchical Sensemaking Framework
PRISMA-ScR: Preferred Reporting Items for Systematic Reviews and Meta-Analyses extension for Scoping Reviews
SDG: Sustainable Development Goals
