# Supplementary material for: Conceptualization From the Sensors to Suicide-Related Outcomes: Scoping Review Based on Layered Hierarchical Sensemaking Framework
Source: JMIR Mhealth Uhealth. 2026 Aug 13;14:e98616. doi: 10.2196/98616 (PMC13472982; doi:10.2196/98616)
Supplement: Supplementary file 1 [file mhealth-v14-e98616-s001.docx]

**Multimedia Appendix 1. Search queries**

This scoping review systematically searched four databases (PubMed, CINAHL, PsycINFO, and IEEE Xplore) to identify studies that examined passively collected digital markers related to suicidality. The search covered publications from January 2015 to December 2025. The search strategy was structured according to the Population–Concept–Context (PCC) framework:

- Population: not restricted
- Concept: passively collected digital markers or indicators derived from smartphones or wearable sensors
- Context: suicide-related mental health outcomes, including suicidal ideation, attempt, or risk

Studies were included if they analyzed passively collected data from smartphones, wearables, or other sensors to identify, monitor, or predict suicide-related outcomes such as suicidal ideation, attempts, or risk. The search terms integrated two major concept groups: (1) suicide-related keywords (suicide, suicidal ideation, suicide risk, suicide prevention, suicide attempt) and (2) digital sensing and phenotyping keywords (smartphone, smartwatch, wearable devices, sensor, passive sensing, mobile sensing, digital marker, digital phenotype). Search syntax and field tags were adjusted according to the requirements of each database. Detailed database-specific search queries are presented below.

**PubMed**

("Suicide"[Mesh] OR "Suicide, Attempted"[Mesh] OR "Suicide Prevention"[Mesh] OR "Suicidal Ideation"[Mesh] OR "Suicidal Behavior"[Mesh] OR suicid*[tiab] OR "suicide risk"[tiab] OR "suicide attempt"[tiab] OR "suicidal thoughts"[tiab])

AND

("Wearable Electronic Devices"[Mesh] OR "Smartphone"[Mesh] OR "Sensors"[Mesh] OR "Mobile Applications"[Mesh] OR "digital marker"[tiab] OR "digital phenotype"[tiab] OR "digital phenotyping"[tiab] OR "mobile sensing"[tiab] OR "smartphone sensing"[tiab] OR wearable*[tiab] OR sensor*[tiab] OR "passive sensing"[tiab] OR "mobile sensor"[tiab] OR "smartwatch"[tiab])

**CINAHL (EBSCOhost)**

(TI suici* OR AB suici* OR TI "suicide risk" OR AB "suicide risk" OR TI "suicidal ideation" OR AB "suicidal ideation" OR TI "suicide prevention" OR AB "suicide prevention" OR TI "suicide attempt*" OR AB "suicide attempt*")

AND

(TI "digital marker" OR AB "digital marker" OR TI "digital phenotype" OR AB "digital phenotype" OR TI "digital phenotyping" OR AB "digital phenotyping" OR TI "mobile sensing" OR AB "mobile sensing" OR TI "smartphone sensing" OR AB "smartphone sensing" OR TI wearable* OR AB wearable* OR TI sensor* OR AB sensor* OR TI "passive sensing" OR AB "passive sensing" OR TI "mobile sensor" OR AB "mobile sensor" OR TI "smartwatch" OR AB "smartwatch")

**PsycINFO**

((DE "Suicide" OR DE "Suicide Risk" OR DE "Suicidal Ideation")

OR

(suicid* OR "suicide risk" OR "suicidal ideation" OR "suicide prevention" OR "suicide attempt*"))

AND

((DE "Mobile Computing" OR DE "Wearable Technology" OR DE "Sensors")

OR

("digital marker" OR "digital phenotype" OR "digital phenotyping" OR "mobile sensing" OR "smartphone sensing" OR wearable* OR sensor* OR "passive sensing" OR "mobile sensor" OR "smartwatch"))

**IEEE Xplore**

("suicide" OR "suicide risk" OR "suicidal ideation" OR "suicide prevention" OR "suicide attempt")

AND

("digital marker" OR "digital phenotype" OR "digital phenotyping" OR "mobile sensing" OR "smartphone sensing" OR wearable OR sensor OR "passive sensing" OR "mobile sensor" OR "smartwatch")
