# Supplementary material for: Conceptualization From the Sensors to Suicide-Related Outcomes: Scoping Review Based on Layered Hierarchical Sensemaking Framework
Source: JMIR Mhealth Uhealth. 2026 Aug 13;14:e98616. doi: 10.2196/98616 (PMC13472982; doi:10.2196/98616)
Supplement: Supplementary file 2 [file mhealth-v14-e98616-s002.docx]

**Multimedia Appendix 2. Operationalization criteria for assigning features to LHSF layers 2 and 3**

| Reference | Low-level feature (Layer 2) | Classification basis for Layer 2 | High-level behavioral marker (Layer 3) | Classification basis for Layer 3 | Layer 3 label: source-defined or author-derived^a^ |
| --- | --- | --- | --- | --- | --- |
| [22] | Linguistic inquiry and word count (LIWC) based word proportions  (e.g., first-person pronouns, emotion-related words) and communication activity  (e.g., message counts and sent-to-received message ratios) | Directly quantifiable linguistic metrics from raw SMS text | Psychological and behavioral patterns  (self-focus, sentiment, social engagement) | Psychosocial constructs inferred by integrating multiple LIWC-derived word proportion and communication activity features through theoretical interpretation of suicidal cognition and affect | Author-derived |
| [23] | Smartphone screen time per app category | Directly quantifiable time metric automatically recorded per app category by passive sensing app | Smartphone use patterns | Behavioral construct inferred by interpreting aggregated per-app screen time data across multiple content categories as an overall pattern of device engagement reflecting behavioral tendencies in media use | Author-derived |
| [24] | High-frequency HRV power | Directly quantifiable physiological index extracted from PPG sensor data via spectral analysis | Reduced parasympathetic activity | Physiological construct inferred from HF HRV values through theoretical interpretation of autonomic nervous system regulation and parasympathetic tone | Source-defined |
| [25] | Number of autonomic events | Directly quantifiable count of EDA-detected skin conductance increases per hour/day, derived by proprietary algorithm from raw EDA sensor data | Autonomic arousal | Physiological construct inferred from aggregated autonomic event counts, interpreted as a marker of sympathetic nervous system activation and psychological distress | Source-defined |
| [26] | Sleep minutes | Directly quantifiable daily average automatically recorded by wristband sensor | Sleep patterns | Behavioral constructs inferred by interpreting aggregated daily sleep minutes and step counts as habitual sleep and activity patterns across the study period | Source-defined |
|  | Step counts |  | Activity patterns |  |  |
| [27] | Incidence of sleep-related text messages,  number of nightly texts sent,  number of unique nightly hour bins | Directly quantifiable counts extracted from raw SMS text data: sleep-related message incidence identified via automated LIWC dictionary matching, and nocturnal texting frequency and spread derived from message timestamps | Sleep disturbance | Behavioral construct inferred by integrating sleep-related linguistic content and nocturnal texting patterns as proxies for wakefulness and sleep disruption through theoretical interpretation of insomnia and sleep disturbance | Source-defined |
| [28] | Root mean square of successive differences (RMSSD), Resting heart rate | Directly quantifiable daily-level metrics derived from sensor data | Distress | Physiological and behavioral constructs inferred from sensor-derived heart rate indices interpreted as markers of psychological distress, step counts interpreted as proxies of withdrawal or low mood, and sleep duration interpreted as an indicator of sleep disruption | Source-defined |
|  | Total steps |  | Physical activity |  |  |
|  | Sleep duration |  | Sleep disturbance |  |  |
| [29] | HRV indices | Directly quantifiable physiological metrics derived from ECG and pressure sensor data | Autonomic imbalance | Physiological construct inferred by interpreting HRV indices and respiratory rate as indicators of sympathovagal dysregulation | Source-defined |
|  | Respiratory rate |  |  |  |  |
| [30] | Distance walked,  time spent at home,  steps taken | Directly quantifiable daily metrics derived from GPS, actigraphy, and app usage logs | Behavioral changes in daily activity profiles | Behavioral construct inferred by integrating daily mobility metrics and app usage into personalized activity profiles and interpreting abrupt transitions between profiles as reflecting clinically meaningful shifts in behavioral patterns | Source-defined |
|  | App usage (binary) |  |  |  |  |
| [31] | Time in bed,  sleep onset latency,  sleep duration,  wake after sleep onset,  sleep efficiency | Directly quantifiable sleep metrics automatically derived from accelerometer data | Sleep disturbance | Behavioral construct inferred by interpreting multiple actigraphy-derived sleep metrics as indicators of disrupted sleep | Source-defined |
| [32] | Time at home,  entropy  (location variability),  distance traveled | Directly quantifiable weekly aggregates derived from GPS data | Social connectedness, behavioral withdrawal,  anhedonia | Psychosocial constructs inferred by interpreting mobility metrics as proxies for clinically meaningful behavioral states | Source-defined |
| [33] | Heart rate,  HRV | Directly quantifiable physiological and behavioral metrics derived from PPG, electrical heart sensors, and accelerometer | Stress states  (Autonomic dysregulation) | HRV indices interpreted as markers of autonomic dysregulation and stress states | Source-defined |
|  | Movement index,  wear-free time,  step count,  walking/running time,  other exercise time,  resting time,  total sleep time |  | Daily behavioral patterns | Behavioral construct inferred by integrating multiple activity and sleep metrics as a composite representation of daily behavioral patterns | Author-derived |
| [34] | Dictionary-based word counts | Directly quantifiable frequency counts of keyword matches extracted from screenshot text via automated dictionary matching | Expression related to suicide risk | Psychological construct inferred by interpreting patterns of suicide-related and general risk language detected across smartphone text as indicators of underlying suicidal cognition and distress | Author-derived |
| [35] | Smartphone screen time  (screenshot image counts, any use, proportion of use) | Directly quantifiable metrics automatically derived from the screen capture app, which recorded screenshots at 5-second intervals during active phone use | Temporal patterns of smartphone use | Behavioral construct inferred by interpreting screen time metrics across proximal versus distal time windows as temporally structured patterns of device engagement | Author-derived |
| Note:  ^a^Source-defined: the original study explicitly labeled the construct. Author-derived: the label was assigned by the review team based on theoretical interpretation. | | | | | |
