# Supplementary material for: Conceptualization From the Sensors to Suicide-Related Outcomes: Scoping Review Based on Layered Hierarchical Sensemaking Framework
Source: JMIR Mhealth Uhealth. 2026 Aug 13;14:e98616. doi: 10.2196/98616 (PMC13472982; doi:10.2196/98616)
Supplement: Supplementary file 3 [file mhealth-v14-e98616-s003.docx]

**Multimedia Appendix 3. Demographic and clinical sample characteristics of the included studies**

| Author  (Year) | Country | Study participants | | | | | | |
| --- | --- | --- | --- | --- | --- | --- | --- | --- |
|  |  | Size (*N*) | Age (Mean±SD, Range) | Female (%) | Setting & General vs. risky | Clinical diagnosis^a^ | Race/Ethnicity (%) | Socioeconomic status |
| Glenn et al.  (2020) [22] | United States | 33 | 20.4 ± 2.4 years (range: not reported) | 84.8% | - Community setting  - High-risk adults who reported a past suicide attempt | Mood disorder (48.5%), Anxiety disorder (39.4%), Substance use disorder (3.0%), Any disorder (57.6%) | - Caucasian (60.6%), Asian (18.2%), African American (9.1%), Multiracial (6.1%), Other (3.0%)  - Not Hispanic (87.9%), Hispanic (12.1%) | Not explicitly reported / College-aged sample (Some college: 94.0%) |
| Coyne et al.  (2021) [23] | United States | 281 | 23.3 ± 1.04 years (range: not reported) | 58% | - Community setting  - General adolescents and emerging adults | Not reported  (general community sample) | Caucasian (80%), Mixed (6%), African American (4%), Asian (4%), Hispanic (3%), Other (3%) | Not explicitly reported /  Educational attainment: high school education or less (7%), some college or currently enrolled (54%), bachelor's degree or higher (38%) |
| Sheridan et al.  (2021) [24] | United States | 51 | 16.7 ± 1.5 years (range: 14–21) | 72.6% | - Emergency department or psychiatric inpatient unit  - High-risk adolescents | Not reported (Characterized by current psychiatric medications)  Antidepressants (68.6%), Antipsychotics (29.4%), ADHD (15.7%), Clonidine (2.0%) | Not reported | Not reported |
| Kleiman et al.  (2021) [25] | United States | 25 | 33.48 ± 13.84 years (range: 19–63) | 56% | - Psychiatric inpatient unit  - High-risk adults | Psychiatric inpatients hospitalized due to suicidal thoughts or behaviors  (specific diagnoses not reported) | White (64%), Asian (20%), Black/African American (8%), Other or multiple ethnicities (8%), Hispanic or Latinx (16%) | Not reported |
| Horwitz et al.  (2022) [26] | United States | 2,881 | 27.6 ± 2.6 years (range: not reported) | 56.8% | - Community setting  - General adults (medical interns) | Not reported  (general community sample) | White (58.0%), Asian (22.7%), Black (4.7%), Hispanic/Latinx (3.8%), Multiracial (8.4%), Other race (2.4%) | Not reported |
| Ladis et al.  (2023) [27] | United States | 26 | 20.8 ± 2.6 years (range: not reported) | 84.6% | - Community setting  - High-risk young adults those reported past suicide attempts | Not reported | White (65.4%), Asian (15.4%), Black (7.7%), Multiple or other (11.5%) | Not reported |
| Czyz et al.  (2023) [28] | United States | 102 | 20.9 ± 2.1 years (range: 18–25) | 81.4% | - Emergency department  - High-risk young adults | Depressive disorder (73.5%), Anxiety disorder (31.4%), Unspecified trauma-and stressor-related disorder (9.8%), Substance use disorder (8.8%), Adjustment disorder (2.9%), Unspecified feeding or eating disorder (2.9%), Unspecified bipolar disorder (2.0%), Unspecified mood disorder (2.0%), Obsessive compulsive disorder (2.0%), Posttraumatic stress disorder (1.0%) | - White (74.5%), More than 1 race (8.8%), Asian (5.9%), Black or African American (4.9%), Other (2.9%), American Indian or Alaska Native (2.0%), Unknown (1.0%)  - Not Hispanic or Latino (87.2%), Hispanic (10.8%), Unknown (2.0%) | Not reported |
| Ortiz et al.  (2024) [29] | Canada | 53 | 44.7 ± 13.1 years (range: not reported) | 66% | - Psychiatric outpatient clinics  - High-risk adults | Bipolar Disorder I (45%) | Caucasian (90%), Hispanic (4%), Asian (4%), Black Canadian (2%) | - Education (years): 15.4±2.7  - Employment status: Work full/part time (49%), On disability (19%), Retired (11%), Student (7%), Home maker (7%), Unemployed (6%) |
| Barrigon et al.  (2023) [30] | Spain | 225 | 43.24 ± 14.13 years (range: not reported) | 62.7% | - Psychiatric outpatient clinic  - High-risk adults | Mood disorders (46.2%), Anxiety disorders (75.1%), Personality disorders (48.4%), Drug abuse (10.7%), Eating disorders (4.4%), Psychotic disorders (0.4%) | Not reported | - Employment status:  Employed or student (44.9%), Unemployed (22.2%), Temporary leave (19.6%), Permanent leave (8%), Retired (5.3%) |
| Mournet et al. (2025) [31] | United States | 7 | 28 ± 10.39 years (range: 19–50) | 71.4% | - Psychiatric outpatient program  - High-risk adults | Borderline personality disorder (100%) | White (71.4%), Asian (14.3%), Hispanic (14.3%) | Not reported |
| Auerbach et al.  (2025) [32] | United States | 186 | 16.43 ± 1.68 years (range: 13–18) | 79.6% | - Psychiatric outpatient programs, emergency departments, or community-based recruitment  - High-risk adolescents | Major Depressive Disorder (60.2%), Bipolar disorder (3.8%), Anxiety disorder (89.8%), Posttraumatic Stress Disorder (15.0%), Substance use disorder (10.8%), Behavioral disorders (Oppositional Defiant Disorder, Conduct disorder, Attention-Deficit/Hyperactivity Disorder) (35.7%) | - White (57.0%), Black or African American (12.4%), More than 1 race (11.3%), Asian (10.2%), American Indian or Alaska Native (0.5%), Unknown or not reported (8.6%)  - Hispanic ethnicity (17.2%) | Annual household income ($):  Less than 25,000 (11.7%), 25,000–100,000 (42.2%), Greater than or equal to 100,000 (46.1%) |
| Um et al.  (2025) [33] | Republic of Korea | 59  (Patient group:  n = 39; healthy controls:  n = 20) | not reported (range: 20–55) | 57.6% | - Psychiatric outpatient clinic; healthy controls recruited via advertisement  - High-risk adults and healthy controls | Major depressive disorder (33.9%), Bipolar disorder (32.2%), Healthy controls (33.9%) | Not reported (Participants were recruited from a medical center in Seoul, Republic of Korea) | - Education years: Patient group (14.8±2.2 years), Healthy controls (16.5±1.7 years)  - Currently having a job: Patient group (66.7%), Healthy controls (89.5%) |
| Ammerman et al.  (2025) [34] | United States | 79 | 35.15 ± 11.07 years (range: 20–63) | 68.3% | - Community setting  - High-risk adults who reported past-month active suicidal ideation or behaviors | Major depressive disorder (43.0%), Generalized anxiety disorder (34.2%), Panic disorder (40.5%), Social anxiety disorder (25.3%), Post-traumatic stress disorder (32.9%), Alcohol use disorder (22.8%), Substance use disorder (25.3%) | - White (84.8%), Black (6.3%), American Indian/Alaska Native (6.3%), Another race (2.6%)  - Non-Hispanic/Latino (92.4%) | Not reported |
| Jacobucci et al.  (2025) [35] | United States | 79 | 35.15 ± 11.07 years (range: 20–63) | 68.3% | - Community setting  - High-risk adults who reported past-month suicidal ideation or behaviors | Major Depressive Disorder (43.0%), Panic Disorder (40.5%), Generalized Anxiety Disorder (34.2%), Post-Traumatic Stress Disorder (32.9%), Social Anxiety Disorder (25.3%), Substance Use Disorder (25.3%), Alcohol Use Disorder (22.8%) | White (84.8%), Non-Hispanic/Latino (92.4%) | - Employment: Employed full-time (44.3%), Unemployed or unable to work (40.5%)  - Education: Completed college graduation (36.7%), Completed some college (27.9%), Completed some high school or a high school degree (18.9%), Completed some advanced education (16.5%) |
| Note.  ^a^ Clinical diagnoses reflect all recorded diagnoses per participant; multiple diagnoses were permitted across studies. | | | | | | | | |
